# Bioelectrical Impedance Analysis Derived-Phase Angle as a Pragmatic Tool to Detect Protein Energy Wasting among Multi-Ethnic Hemodialysis Patients

**DOI:** 10.3390/diagnostics11101745

**Published:** 2021-09-23

**Authors:** Cordelia-Kheng-May Lim, Jun-Hao Lim, Imliya Ibrahim, Yoke-Mun Chan, Nor Fadhlina Zakaria, Rosnawati Yahya, Zulfitri Azuan Mat Daud

**Affiliations:** 1 Department of Dietetics, Faculty of Medicine and Health Sciences, Universiti Putra Malaysia, UPM Serdang 43400, Malaysia; cordeliamay07@gmail.com (C.-K.-M.L.); sinhao0624@yahoo.com (J.-H.L.); imliya.ibrahim@gmail.com (I.I.); cym@upm.edu.my (Y.-M.C.); 2 Research Center of Excellent (RCoE) Nutrition and Non-communicable Diseases, Faculty of Medicine and Health Sciences, Universiti Putra Malaysia, UPM Serdang 43400, Malaysia; 3 Department of Dietetics, Hospital Pengajar Universiti Putra Malaysia, UPM Serdang 43400, Malaysia; 4 Department of Medicine, Faculty of Medicine and Health Sciences, Universiti Putra Malaysia, UPM Serdang 43400, Malaysia; n_fadhlina@upm.edu.my; 5 Department of Nephrology, Hospital Kuala Lumpur, Kuala Lumpur 50586, Malaysia; rosnayahya@gmail.com

**Keywords:** phase angle, bioelectrical impedance, body composition, protein energy wasting, hemodialysis

## Abstract

Protein-energy wasting (PEW) is a devastating metabolic derangement that leads to increased morbidity and mortality in hemodialysis (HD) patients. This study aimed to determine the diagnostic test accuracy of bioelectrical impedance analysis derived-phase angle (PhA) in detecting PEW among HD patients. This was a multi-centre, cross-sectional study conducted amongst 152 multi-ethnic HD patients in Klang Valley, Malaysia. PEW was assessed using the International Society of Renal Nutrition and Metabolism criteria as the reference method. PhA was measured using a multi-frequency bioelectrical impedance spectroscopy at 50 kHz. Multiple and logistic regressions were used to determine factors associated with PhA and PEW diagnosis, respectively. A receiver operating characteristics curve analysis was used to establish the gender-specific PhA cut-offs to detect PEW. PEW existed in 21.1% of the HD patients. PhA was found as an independent predictor of PEW (adjOR = 0.308, *p* = 0.001), with acceptable to excellent discriminative performance (adjAUC_male_ = 0.809; adjAUC_female_ = 0.719). Male patients had higher PhA cut-off compared to female patients (4.26° vs. 3.30°). We concluded that PhA is a valid and pragmatic biomarker to detect PEW in multi-ethnic Malaysian HD patients and a gender-specific cut-off is necessary, attributed to the gender differences in body composition.

## 1. Introduction

In the 21st century, the nutrition paradigm has been shifting towards combating protein energy wasting (PEW) in the HD population [1]. PEW is a maladaptive metabolic state in which both body protein mass and energy fuel reserves are depleted [2]. PEW is prevalent in patients with chronic kidney disease (CKD) and worsens over time as the disease progresses (from < 5% in CKD Stage 1–2 up to 11–54% in CKD Stage 3–5) [3,4,5]. A meta-analysis reported that the global prevalence of PEW among dialysis patients ranged from 28.0% to 54.0% [5]. The etiology of PEW is multifactorial, namely including decreased dietary intake, inflammation, metabolic derangements, comorbidities, and dialysis treatment [6]. The consequences of PEW include weakness, poor quality of life, increased risk of hospitalisation, and heightened morbidity and mortality [7,8]. The total annual cost for end-stage renal disease (ESRD) expenditure was reported to be approximately 100,000 USD per patient [9]. On top of that, there has been considerable cost required for the healthcare expenses of ESRD patients with PEW due to higher rates of hospitalisation and recurrent visits to the emergency department or outpatient clinics [10]. Hence, the presence of PEW in the ESRD population would further worsen the global economic burden.

Regular assessment is crucial to detect PEW in these vulnerable populations for better nutritional outcomes and survival. The International Society of Renal Nutrition and Metabolism (ISRNM) expert panel have proposed a set of criteria to diagnose PEW in CKD patients [2]. Nonetheless, the ISRNM criteria are subjected to several constraints that limit their application in the clinical setting [11]. For instance, it requires multi-dimensional parameters to diagnose PEW, such as (i) body mass, (ii) serum chemistry, (iii) muscle mass, and (iv) dietary intake. These assessments demand well-trained personnel (i.e., dietitians) and can be cumbersome for them to perform due to time constraints. This creates a great challenge, especially in low- and middle- income countries where a dietitian shortfall (20–45%) puts these countries in hot water [9].

As such, a bioelectrical impedance analysis (BIA)-derived phase angle (PhA) has been emerging as a valid proxy of PEW by assessing the nutritional state at the cellular level [11,12,13,14,15]. PhA is derived from the arc tangent value of the ratio of reactance (Xc) to resistance (R) [16]. Reactance denotes cell membrane integrity, in which healthy cells have a more intact cellular membrane [17]. On the other side of the coin, resistance is the opposition to electrical current, and is inversely proportional to the amount of lean muscle mass in the body [17,18]. Therefore, PhA is considered an indicator of cellular health [19]. A higher PhA value is more favourable as it denotes better cellular function and life expectancy [20,21].

Mounting evidence has supported the use of PhA as a prognostic indicator for various clinical conditions (i.e., cancer, cirrhosis, surgery, and frailty) [19,22,23,24]. Although PhA has also been proposed as a rapid, inexpensive, and non-invasive method to detect PEW among dialysis patients in several countries (i.e., China, Japan, Mexico, and Spain) [11,12,13,14,15], the extrapolation of their findings is questionable due to the variation in body composition across ethnicities [25] as evidenced by the inconsistent cut-offs identified (ranging from 3.7° to 4.64°) [11,12,13,14,15]. In addition, the absence of gender-specific cut-offs for PhA might diminish its diagnostic accuracy for PEW detection. Therefore, in this present study, we aimed to: (i) examine the diagnostic test accuracy of PhA, and (ii) to establish optimal gender-specific PhA cut-offs to detect PEW among multi-ethnic Malaysian HD patients.

## 2. Materials and Methods

### 2.1. Study Design and Patient Recruitment

A cross-sectional study was conducted among multi-ethnic HD patients residing in the Klang Valley, Malaysia. Subject recruitment commenced on February 2019 up to July 2019 at 9 conveniently selected HD centres, consisting of dialysis units in a tertiary government hospital, non-government organisations and private HD centres (within a 40 km radius from the Faculty of Medicine and Health Sciences, Universiti Putra Malaysia). Patients were eligible for participation if they were at least 18 years old and undergoing regular HD treatment 3 times per week for at least 6 months prior to the data collection. Patients were excluded if they had: (i) contraindications for BIA measurement (i.e., limb amputations, metallic implants, or having a pacemaker); (ii) visual, hearing or speech impairment; (iii) acquired immunodeficiency syndrome, malignancy, or underwent surgery that interfered with their nutritional status for the past 3 months; (iv) frailty or severely ill; (v) involved in a clinical trial; and (vi) cognitive impairment such as Alzheimer’s or mental illness. This study was approved by the Ethics Committee of the National Medical Research Register, Ministry of Health, Malaysia (NMRR-18-1514-42126, approved on 18 September 2018)) and the Universiti Putra Malaysia Ethic Committee for Research Involving Human Subjects (JKEUPM-2019-064, approved on 1 February 2019). Both written and verbal informed consent were obtained from all the patients prior to their study participation. All research procedures were conducted in accordance with the World Medical Association-Declaration of Helsinki. The study sections were reported according to the Strengthening the Reporting of Observational Studies in Epidemiology (STROBE) checklist [26].

### 2.2. Sample Size Requirement and Sampling Method

Sample size was calculated using G. Power version 3.1.9.4 (Franz Faul, Universitat Kiel, Germany) statistical software for a Linear Multiple Regression: Fixed Model, R^2^ deviation from zero, with an effect size of 0.15, statistical power of 80%, 5% level of significance, and with a total of 16 predictors identified from previous studies [11,12,13,18,27,28]. The calculated sample size was 143 patients. After accounting for a 30% non-response rate, a total of 204 patients were recruited. A quota sampling method was used to recruit an equal proportion of male and female (102 each) HD patients.

### 2.3. Research Instrument

A semi-structured questionnaire was used to collect the patients’ information including: (i) sociodemographic data; (ii) clinical data; (iii) BIA measurement; (iv) anthropometric measurements; (v) biochemical data; (vi) dietary intake assessment; and (vii) PEW diagnosis.

### 2.4. Sociodemographic Data and Clinical Data

Sociodemographic background (i.e., age, gender, ethnicity, marital status, education level, employment status, as well as monthly household income) were obtained via face-to-face interview. Clinical data (i.e., year diagnosed with ESRD, dialysis vintage, presence of comorbidities, and blood pressure) were retrieved from both paper-based and electronic medical records at the respective HD centres.

### 2.5. Nutritional Status Assessments

#### 2.5.1. Bioelectrical Impedance Analysis Measurement (Index Test)

In this study, PhA derived from BIA measurement was the index test [29]. BIA measurement was performed using a simple and portable multi-frequency (5–1000 kHz) whole-body bioimpedance spectroscopy (BIS) (BCM, Fresenius Medical Care, Bad Homburg, Germany). The output parameters of this BIS device have been validated against gold standard reference methods of body composition measurements in previous studies [30]. Measurements were conducted before patients’ HD treatment as per the manufacturer’s guidelines [30] during the midweek dialysis session. This is because the ultrafiltration process during a dialysis session can affect fluid distribution in the body, which in turn has significant effects on the whole-body impedance measurements [31,32]. Patients were required to place themselves in a posterior recumbent position with disposable electrodes placed on 4 contact areas (i.e., metacarpophalangeal joint, wrist, metatarsophalangeal joint, and ankle) at the non-fistula access site (Supplementary Material Figure S1). The entire BIA measurement process took approximately 5 min. A measurement quality value of ≥90% (a smooth, dome shape of a Cole–Cole plot) indicates a successful measurement [33,34]. The PhA value [arctangent (Xc/R) × (180/π)], expressed in degree (°) was obtained from the BIA reading measured at the frequency of 50 kHz, in which the maximum reactance occurs, whereby the body cells are strongest in resisting the current, providing the highest PhA value [35]. The other BIA derived-parameters: body fat percentage (BF%), measurement quality, overhydration (OH), lean tissue index (LTI), fat tissue index (FTI), intracellular water (ICW), extracellular water (ECW), and body cell mass (BCM) were also recorded.

#### 2.5.2. Anthropometric Measurements

Height, weight, mid-arm circumference (MAC), and triceps skinfold thickness (TSF) were measured in accordance with the International Society for the Advancement of Kinanthropometry (ISAK) by trained research dietitians [36]. Patients’ heights were measured using a portable stadiometer (SECA-213, Hamburg, Germany), whereas pre- and post-dialysis weight was measured using the weighing scale available at the respective dialysis units. In addition, post-dialysis weights during the past 3 months were retrieved from the patient’s dialysis book to assess the extent of unintentional weight loss. Body mass index (BMI) was calculated using the formula of post-dialysis weight (kg)/height (m^2^). MAC was measured using a non-extensible, Lufkin metal measuring tape (Apex Tool Group, LLC, Sparks, NC, USA), while TSF was measured using a Harpenden skinfold calliper (HSK-BI, British Indicators, West Sussex, UK). Both the MAC and TSF measurements were performed on the non-fistula arm of the patient. Mid-arm muscle circumference (MAMC) and corrected mid-arm muscle area (MAMA) for gender were then calculated using the following equations [37,38]:

MAMC (cm) = [MAC (cm) − π × TSF (cm)] MAMA (male) = [(MAC (cm) − π × TSF (cm))^2^/4π] − 10 MAMA (female) = [(MAC (cm) − π × TSF (cm))^2^/4π] − 6.5

All measurements were taken twice, and the mean value was recorded. A third measurement was obtained when the difference between the first and second measurement exceeded 5% for the TSF measurement, or 1% for other measurements (i.e., height, weight, and MAC), as outlined in the ISAK protocol [36].

#### 2.5.3. Biochemical Data

Patients’ biochemical data (i.e., pre- and post-dialysis serum urea, pre-dialysis serum creatinine, serum albumin, and serum cholesterol) were obtained based on the latest routine blood result (i.e., within a month). Fasting blood samples were collected by the dialysis nurse prior to their dialysis session. Blood samples were then analysed by the respective in-house hospital laboratories and external certified laboratories. Dialysis treatment adequacy (Kt/V), which measures urea removal during a patient’s dialysis treatment, was calculated using the Daugirdas formula [39].

#### 2.5.4. Dietary Intake Assessment

Diet records were collected for 3 days (1 dialysis day, 1 non-dialysis day, and 1 optional weekend) by trained research dietitians [38]. Standard household measurement tools were used to guide patients in estimating food and beverage portions. The total dietary energy intake (DEI) and dietary protein intake (DPI) were analysed using Nutritionist Pro Software version 4.0.0 (Axxya Systems, LLC, Stafford, TX, USA). The Nutrient Composition of Malaysian Foods [40], and Energy and Nutrient Composition of Food, Singapore, [41] were the main sources of food database references for food data entry into the software. The adequacy of energy and protein intake were interpreted based on the patient’s ideal body weight (IBW) or adjusted body weight if the patient’s weight was <95% or >115% of the IBW [42]. Implausible reported energy intake was determined using the Goldberg cut-off based on the ratio of the reported energy intake to the basal metabolic rate (EI_rep_:BMR) [43]. Patients’ BMR was estimated using the Harris-Benedict equation [44]. A physical activity level of 1.3 was applied for BMR calculation [45]. Patients having an EI:BMR of <0.8, 0.8–2.0, and >2.0 were classified as under-reporters, acceptable reporters, and over-reporters of energy intake, respectively.

#### 2.5.5. PEW Diagnosis according to the ISRNM Criteria (Reference Standard)

The reference standard denotes the best available method to detect patients who have the outcome of interest [29]. Due to lack of a gold standard method to detect PEW among HD patients, the criteria proposed by the ISRNM expert panel served as the reference standard for PEW diagnosis among the HD patients. It consists of 4 main criteria: (i) body mass (BMI < 23 kg/m^2^; BF% < 10%; unintentional weight loss over time: 5% over 3 months or 10% over 6 months), (ii) muscle mass (MAMC: reduction > 10% in relation to 50th percentile of reference population; reduced muscle mass: 5% over 3 months or 10% over 6 months), (iii) serum chemistry (serum albumin < 38 g/L; serum cholesterol < 2.59 mmol/L), and (iv) dietary intake (unintentional low DPI < 0.8 g/kg BW/day; unintentional low DEI < 25 kcal/kg BW/day) [2]. Patients are diagnosed with PEW if they fulfil at least 3 out of the 4 criteria (at least 1 component in each of the listed criteria) [2]. The criteria proposed by ISRNM for PEW diagnosis are listed in the Supplementary Materials (Table S1).

### 2.6. Statistical Analyses

Data were analysed using the Statistical Package for Social Sciences (SPSS) software version 26.0 (IBM, Chicago, IL, USA). Continuous variables with normal distribution were presented as mean ± standard deviation (SD), whereas skewed data were presented as median (q1–q3). Categorical variables were expressed in frequency (*n*) and percentages (%). Normality assumption was checked using the Shapiro–Wilk test and visual inspection of the histogram.

There were a total of 8 variables, with missing data ranging from 1.3% to 9.9% for MAC (*n* = 2, 1.3%), TSF (*n* = 3, 2.0%), pre-dialysis serum urea (*n* = 3, 2.0%), serum albumin (*n* = 4, 2.6%), post-dialysis serum urea (*n* = 5, 3.3%), current pre-dialysis serum creatinine (*n* = 6, 3.9%), serum cholesterol (*n* = 7, 4.6%), and past-3 months pre-dialysis serum creatinine (*n* = 15, 9.9%). Missing data were handled using multiple imputation methods to provide unbiased estimates of the missing values, as well as to preserve the sample size required to achieve sufficient statistical power [46]. A total of 10 iterations were imputed [47] and the mean value was obtained to replace the missing values.

The Pearson product-moment correlation was used to determine the magnitude and direction of the bi-variate relationships between PhA with PEW criteria and body composition. An independent t-test and one-way ANOVA were used to examine the mean differences in PhA across patients’ characteristics. Hierarchical multiple linear regression (MLR) was used to determine the predictors of PhA. Variables were entered sequentially into two blocks, Block 1: patients’ characteristics, and Block 2: Block 1 + nutritional parameters. Subsequently, logistic regression was performed to determine the odds ratio for PhA according to the PEW criteria.

A receiver-operating characteristics (ROC) curve analysis was used to determine diagnostic accuracy and establish the gender-specific cut-offs for PhA to detect PEW. The area under the curve (AUC) indicates the discriminative power of the test. An AUC of 0.5 indicates no discriminative power, >0.5 to <0.7 indicates poor discriminative power, 0.7 to <0.8 indicates acceptable discriminative power, ≥0.8 to <0.9 indicates excellent discriminative power, and ≥0.9 indicates outstanding discriminative power [48]. Statistical significance was set at *p* < 0.05.

## 3. Results

### 3.1. Patient Recruitment

A total of 310 patients were approached but only 236 patients were eligible to participate in the study. The reasons for exclusion were: (i) dialysis vintage < 6 months (*n* = 37), (ii) frail and severely ill (*n* = 25), (iii) visual impairment (*n* = 4), (iv) speech impairment (*n* = 2), and (v) involved in clinical trial (*n* = 6). Out of the 236 eligible patients, a total number of 204 patients were successfully recruited (response rate = 86.4%). Prior to final data analysis, 52 patients were excluded, attributable to energy misreporters (*n* = 18), missed BIA measurements (*n* = 11), and BIA measurement quality < 90% (*n* = 23), resulting in a final number of 152 patients. The flowchart for patient screening and recruitment is as depicted in the Supplementary Materials (Figure S2).

### 3.2. Patients’ Characteristics

The sociodemographic and clinical data are summarised Table 1. The median age of the patients was 58.5 (50.0–65.8) years, ranging from 25 to 77 years old. There were 81 (53.3%) males and 71 (46.7%) females. The major ethnic group composition consisted of Malays (55.3%), followed by Chinese (32.9%), and Indians (11.8%), which is a similar ethnic composition to the HD population in Klang Valley [49]. The majority of the patients received a secondary education (46.7%), were married (89.5%), unemployed (74.3%), and had a monthly income of >RM1000 @ USD 237.71 (50.7%). Hypertension (75.7%), diabetes mellitus (34.9%), and hyperlipidemia (30.9%) were the three major comorbidities that co-existed among the HD patients. More than half (56.6%) of the HD patients had at least two comorbid conditions. The median dialysis vintage was 56 (30.0–97.8) months. The majority of the patients were adequately dialysed (84.2%) as indicated by the mean Kt/V of 1.5 ± 0.3.

### 3.3. Comparison of PhA across Patients’ Characteristics

The comparisons of PhA across patients’ characteristics are depicted in the Supplementary Materials (Table S2). A significant lower PhA was observed in patients who are older, Chinese, have a lower education level and are unemployed (*p* < 0.05). Male patients had a significantly higher PhA compared to females (4.62 ± 0.82° vs. 3.92 ± 0.88°; *p* < 0.001). Patients who have ≥3 comorbidities were also seen to have a significantly lower PhA compared to those with only one comorbidity (4.03 ± 0.86° vs. 4.59 ± 0.96°; *p* = 0.020).

### 3.4. Correlations between PhA with PEW Criteria and Body Composition in HD Patients

PhA was significantly correlated with the majority of the PEW sub-components according to the ISRNM (*p* < 0.05) (Supplementary Materials Table S3). For instance, PhA has a strong positive correlation with serum creatinine (*r* = 0.542, *p* < 0.001), a moderate positive correlation with MAMC (*r* = 0.444, *p* < 0.001), and a weak positive correlation with serum albumin (*r* = 0.283, *p* < 0.001), BMI (*r* = 0.175, *p* = 0.031), and serum cholesterol (*r* = 0.209, *p* = 0.010). Contrarily, PhA has a moderate negative correlation with BF% (*r* = −0.382, *p* < 0.001). However, no significant correlation was observed between PhA with unintentional weight loss (*r* = 0.093, *p* = 0.255), DEI (*r* = 0.074, *p* = 0.362) and DPI (*r* = 0.057, *p* = 0.484). In addition, PhA was significantly correlated with body composition parameters (*p* < 0.05) (Supplementary Materials Table S3). For instance, PhA has a strong positive correlation with a LTI (*r* = 0.718, *p* < 0.001), ICW (*r* = 0.658, *p* < 0.001), and BCM (*r* = 0.690, *p* < 0.001), and a weak positive correlation with ECW (*r* = 0.251, *p* = 0.002). On the other hand, PhA has a moderate negative correlation with OH (*r* = −0.420, *p* < 0.001) and a weak negative correlation with a FTI (*r* = −0.160, *p* = 0.048).

### 3.5. Predictors of PhA in HD Patients

The hierarchical MLR analysis results are presented in Table 2. In Block 1, patients’ characteristics accounted for a significant 39.4% of the variance in PhA (R^2^ = 0.394, F (12, 139) = 7.527, *p* < 0.001). PhA could be predicted by age (*β* = −0.395, *p* = < 0.001), gender (*β_female_* = −0.374, *p* < 0.001), ethnicity (*β_Indian_* = −0.189, *p* = 0.010), and dialysis vintage (*β* = −0.154).

In Block 2, the addition of nutritional markers accounted for an additional 20.8% of variance in PhA (**Δ**R^2^ = 0.208, **Δ**F (9, 130) = 7.565, *p* < 0.001). BMI (*β* = 0.266, *p* = 0.018), BF% (*β* = −0.334, *p* < 0.001), pre-dialysis serum creatinine (*β* = 0.229, *p* = 0.003), serum albumin (*β* = 0.205, *p* = 0.001), and serum cholesterol (*β* = 0.171, *p* = 0.005) were significant predictors of PhA. Age (*β* = 0.199, *p* = 0.017) remained as a significant independent predictor of PhA after adjusting for nutritional markers. On the other hand, the significant effects of gender, ethnicity, and dialysis vintage were lost after the addition of nutritional markers into the model (*p* > 0.05). In summary, PhA in HD patients could be predicted based on the regression model equation as stated: PhA = 0.435 − (0.016 × age in years) + (0.056 × BMI) + (−0.034 × BF%) + (0.001 × pre-dialysis serum creatinine) + (0.054 × serum albumin) + (0.153 × serum cholesterol).

### 3.6. Associations of PhA and PEW Criteria in HD Patients

**Figure 1:**
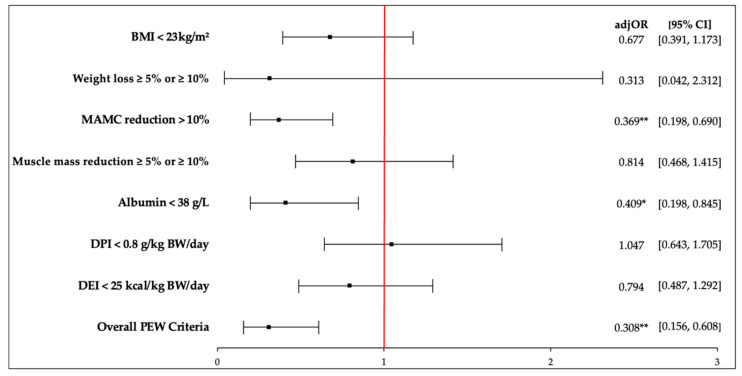
Forest plot for adjusted odds ratio of PhA as per PEW criteria according to ISRNM. Abbreviations: adjOR, adjusted odds ratio; BMI, body mass index; MAMC, mid-arm muscle circumference; DPI, dietary protein intake; DEI, dietary energy intake; PEW, protein energy wasting. OR was adjusted for age, gender, ethnicity, education level, marital status, employment, monthly income, comorbidities, dialysis vintage, and Kt/V. The red line represents odds ratio value of 1 (no association). Serum cholesterol and body fat percentage were not included as the expected frequency had less than five representative cases. * *p* < 0.05, ** *p* < 0.01.

PEW was evident in 21.1% of the HD patients as per the ISRNM criteria. The adjusted odds ratio for PhA, as per the PEW criteria, is depicted in Figure 1. Individual PEW criteria were dichotomised according to the cut-off proposed by the ISRNM. PhA is a significant predictor of PEW after adjusting for patients’ characteristics (adjOR: 0.308, 95% CI = 0.156, 0.608, *p* = 0.001). Patients with lower PhA had 3.2 times higher odds to be diagnosed with PEW (met at least 3 out of the 4 criteria) (adjOR = 0.308, 95% CI = 0.156, 0.608). However, only 2 out 7 of the PEW criteria examined could be significantly predicted by PhA, namely MAMC reduction > 10% (*p* = 0.002) and serum albumin < 38 g/L (*p* = 0.016). An increase in one unit of PhA could significantly reduce the odds of having serum albumin < 38 g/L by 59.1% (adjOR = 0.409, 95% CI = 0.198–0.845), followed by a 63.1% reduction in the odds of having a MAMC reduction > 10% (adjOR = 0.369, 95% CI = 0.198–0.690).

### 3.7. PhA Cut-Offs to Detect PEW in HD Patients

**Figure 2:**
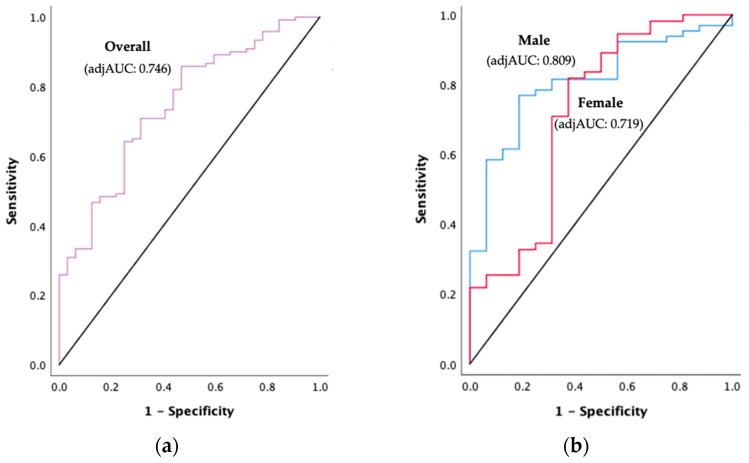
(**a**) Overall ROC curve analysis for PhA to detect PEW among HD patients; (**b**) ROC curve analysis for PhA according to gender to detect PEW among HD patients. Abbreviations: ROC, receiver operating characteristics; adjAUC, area under the curve adjusted for age, dialysis vintage, comorbidities, measurement quality, and overhydration.

PhA was significantly lower in the PEW group (3.75° ± 0.90) compared to the non-PEW group (4.43° ± 0.87) (*p* < 0.001). The PhA cut-offs for detecting PEW and their diagnostic accuracy measures are depicted in Figure 2 and Table 3. ROC curve analysis showed that PhA had a significant acceptable to excellent discriminative performance in detecting PEW among HD patients (adjAUC_overall_ = 0.746, *p* < 0.001; adjAUC_male_ = 0.809, *p* < 0.001; adjAUC_female_ = 0.719, *p* = 0.007). The overall PhA cut-off for PEW diagnosis was 4.11° (sensitivity = 62.5%, specificity = 61.7%). On the other hand, the PhA cut-off for PEW diagnosis in male patients was 4.26° (sensitivity = 68.8%, specificity = 67.7%), whereas female patients had a lower PhA cut-off at 3.30° (sensitivity = 68.8%, specificity = 85.5%). The overall model quality was >0.5, representing a good model prediction as shown in the Supplementary Materials (Figure S4).

## 4. Discussion

This study provides a comprehensive view of knowledge pertaining to patients’ characteristics using PhA and its prediction for PEW diagnosis. As shown in this study, PhA declined with aging, and this might be due to the profound age-related changes in body composition including skeletal muscle loss (reactance) [50] and fat mass accumulation (resistance) [20]. Furthermore, our study also suggests that the deterioration of PhA (i.e., cell membrane integrity) might also occur due to the effect of aging per se [51], irrespective of body composition changes (see Block 2 in Table 2). In line with previous studies, the differences in PhA across gender, ethnicity, and dialysis vintage were also found to be confounded by body composition (see Block 2 in Table 2) [18,25,52].

Despite the PhA disparity across patients’ characteristics in different populations [11,12,13,15], the external validity of PhA in detecting PEW among the HD population has been extended to a multi ethnicity population. In this study, PhA was found to be an independent predictor of PEW in HD patients (adjOR: 0.308, *p* = 0.001). As expected, patients with PEW had a lower PhA compared to their non-PEW counterparts [11,15]. PEW is a pathological condition accompanied with depletion of fat and muscle stores the body [2] which disrupts the normal function of healthy cells by altering their membrane integrity and function [20]. Surprisingly, although PhA correlated with the majority of the nutritional markers, it failed to predict most of the PEW criteria using the ISRNM cut-offs. Since the ISRNM cut-offs are derived from the American population, its utility in other countries has been disputed [53]. For instance, there was a drastic difference in the number of HD patients diagnosed with PEW using the ISRNM criteria (13.3%) compared to the Subjective Global Assessment (80.0%) [54]. In this study, only 0.7% (1/152) of the HD patients fulfilled the criteria of body fat percentage < 10%, and 2.0% (3/152) fulfilled the criteria of having serum cholesterol < 2.59 mmol/L. This might reflect the unequal contribution of individual ISRNM criterion towards a PEW diagnosis, and imply the need to revisit the use of these cut-offs in non-Caucasians. Interestingly, PhA seems to be less sensitive to reflect dietary energy and protein intake in the current study. This relationship could be attenuated owing to regression dilution bias because of day-to-day variation in dietary intake [55,56]. However, this did not confiscate the discriminative ability of PhA to detect PEW among HD patients in both current and previous studies [12,15].

The optimal PhA cut-off (4.11°) to detect PEW among multi-ethnic HD patients in this study was within the range of values found in previous studies. Nonetheless, it is worth noting that a wide range in PhA cut-offs were reported (3.7–4.64°) [11,12,13,14,15]. This could be due to a number of reasons including the differences in age distribution, gender ratio, ethnic groups, as well as the type of BIA device used (Supplementary Materials Table S4). For instance, the PhA cut-off proposed by Leal-Escobar et al. was the highest (4.64°) compared to other studies, and this could be explained by the relatively lower body fat percentage and higher muscle mass percentage of the Western nation compared to Asian [11,57]. Correspondingly, sub-group analysis in this study demonstrated ethnic differences in PhA cut-offs for a PEW diagnosis (Supplementary Materials Table S5). Furthermore, previous studies were conducted among populations with vast age ranges (i.e., mean age of 36.5 to 68.0 years old in the literature) [11,12,13,14,15] and this might contribute to the variation in PhA. In addition, the measurement frequency of the BIA device used (e.g., 5, 50, or 250 kHz) also affects PhA [58]. Although multi-frequency measurements show a better ability to estimate extracellular fluid volume (i.e., hydration status) than single-frequency measurement, good agreement was found for intracellular fluid volume (i.e., nutritional status) [59]. To date, 50 kHz is ideal for PhA measurements [35]. Therefore, healthcare professionals should consider the type and measurement frequency of BIA devices based on the purpose of assessment. Furthermore, this study implies the need for gender-specific PhA cut-offs to detect PEW in HD patients, attributed to the difference in fat and muscle mass composition. The current study found that males have a higher PhA cut-off (4.26°) compared to females (3.30°). This is because males have a higher proportion of muscle mass (reactance) and lower body fat percentage (resistance) compared to females with the same BMI [60].

The sensitivity (68.8%) of the gender-specific PhA cut-off found in this study was slightly lower compared to previous studies (ranging from 77.7% to 86.4%) [11,13,15]. This could be due to the different methods used to select the most optimal PhA cut-off point on the ROC curve [61], which has not been reported in previous studies. In this study, both sensitivity (68.8%) and specificity (ranging from 67.7% to 85.5%) indices were maximised to correctly identify those patients with PEW (true positive) and without PEW (true negative) [61]. Notably, specificity was prioritised in this study to minimise false-negative results which can cause detrimental outcomes due to misdiagnosis.

This study was subjected to several limitations that could be addressed in forthcoming studies. In light of the cross-sectional study design, the predictive validity of PhA to diagnose PEW cannot be established. Thus, longitudinal predictive research could be employed to strengthen the validity of the study findings. Moreover, a non-probability sampling method was used, hence, the results of this study could not be generalised to the entire Malaysian population. The predictive accuracy of PhA might be slightly diminished by age, gender, and ethnic differences in body composition. Therefore, future studies should consider establishing age-, ethnicity-, and gender-specific PhA cut-offs to further improve on the predictive accuracy. In addition, inter-device validation studies are also required to determine the agreement in PhA measurements for PEW diagnosis in HD patients. Despite the limitations, this study provides the evidence to support the use of PhA to detect PEW in multi-ethnic HD populations. Furthermore, we also provide novel insights on the relevance of using gender-specific PhA cut offs for PEW diagnosis in HD patients.

## 5. Conclusions

PhA appears as a pragmatic and valid biomarker which allows for the rapid detection of PEW among multi-ethnic HD patients in a clinical setting. PhA cut-offs, at 4.26° for male and 3.30° for female, had acceptable to excellent discriminative performance in detecting PEW among multi-ethnic HD patients. Healthcare professionals should consider the use of PhA measurements in making data-driven decisions to improve the quality of patient care.

## Figures and Tables

**Table 1:** Patients’ Characteristics (*n* = 152).

Variables	*n* (%)	Median (q1–q3)	Range
Age (years)		58.5 (50.0–65.8)	25–77
Gender			
Male	81 (53.3)		
Female	71 (46.7)		
Ethnicity			
Malay	84 (55.3)		
Chinese	50 (32.9)		
Indian	18 (11.8)		
Education level			
Primary	42 (27.6)		
Secondary	71 (46.7)		
Tertiary	39 (25.7)		
Marital status			
Single	16 (10.5)		
Married	136 (89.5)		
Employment			
Employed	39 (25.7)		
Unemployed	113 (74.3)		
Monthly income			
≤RM1000	75 (49.3)		
>RM1000	77 (50.7)		
Comorbidities ^a^			
Hypertension	115 (75.7)		
Diabetes mellitus	53 (34.9)		
Hyperlipidemia	47 (30.9)		
Others ^b^	41 (27.0)		
No of comorbidities			
None	15 (9.9)		
One	51 (33.6)		
Two	47 (30.9)		
≥Three	39 (25.7)		
Dialysis vintage (months)		56 (30.0–97.8)	6–272
Dialysis adequacy (Kt/V)		1.5 ± 0.3 ^c^	0.6–2.5
Adequate (≥1.2)	128 (84.2)		
Inadequate (<1.2)	24 (15.8)		

Data are expressed as *n* (%) or median (q1–q3). ^a^ The sum of frequency for comorbidities exceeds the total number of HD patients because some patients have multiple comorbidities; ^b^ Other major comorbidities denote cardiovascular disease, hepatitis B & C, stroke, gastritis, asthma, gout, and hypothyroidism (refer to Supplementary Materials Figure S3 for more details); ^c^ Continuous data with normal distribution are expressed as the mean ± SD.

**Table 2:** Predictors of PhA in HD Patients (*n* = 152).

Variables	Model 1			
	Block 1		Block 2	
	*β*	R^2^	*β*	R^2^
Age	−0.395 ***	0.394	−0.199 *	0.602
Gender ^a^				
Female	−0.374 ***		−0.090	
Ethnicity ^b^				
Chinese	−0.058		−0.050	
Indian	−0.189 *		−0.044	
Education level ^c^				
Secondary	0.047		−0.014	
Tertiary	−0.023		−0.028	
Marital status ^d^				
Married	0.039		−0.007	
Employment ^e^				
Unemployed	−0.079		−0.054	
Monthly income ^f^				
≤RM 1000	0.060		0.051	
Clinical data				
No. of comorbidities	−0.101		−0.079	
Dialysis vintage (months)	−0.154 *		−0.089	
Dialysis adequacy (Kt/V)	−0.011		0.049	
Body mass				
BMI (kg/m^2^)	-		0.266 *	
BF (%)	-		−0.334 ***	
Unintentional weight loss (%)	-		0.007	
Muscle mass				
MAMC (cm)	-		0.111	
Serum creatinine (umol/L)	-		0.229 **	
Serum chemistry				
Albumin (g/L)	-		0.205 **	
Cholesterol (mmol/L)	-		0.171 **	
Dietary intake				
DEI (kcal/kg BW/day)	-		0.025	
DPI (g/kg BW/day)	-		0.036	

Reference group: ^a^ Male, ^b^ Malay, ^c^ Primary education, ^d^ Single, ^e^ Employed, ^f^ Monthly income > RM 1000. Abbreviations: BMI, body mass index; BF, body fat; MAMC, mid-arm muscle circumference; DEI, dietary energy intake; DPI, dietary protein intake. Model 1: Hierarchical Multiple Regression Model for PhA; Block 1: Patients’ characteristics; Block 2: Block 1 + nutritional parameters; * *p* < 0.05, ** *p* < 0.01, *** *p* < 0.001.

**Table 3:** Diagnostic Accuracy of PhA to detect PEW in HD patients (*n* = 152).

	PhA Cut-Off (°)	adjAUC	Sensitivity (%)	Specificity (%)	*p*-Value
Overall (*n* = 152)	4.11	0.746	62.5	61.7	<0.001
Male (*n* = 81)	4.26	0.809	68.8	67.7	<0.001
Female (*n* = 71)	3.30	0.719	68.8	85.5	0.007

Abbreviations: PhA, phase angle; adjAUC, area under the curve adjusted for age, dialysis vintage, comorbidities, measurement quality, and overhydration. Data was analysed using Receiver Operating Characteristics curve analysis.

## Data Availability

The data presented in the study are available on request from the corresponding author.

## Supplementary Materials

The following are available online at https://www.mdpi.com/article/10.3390/diagnostics11101745/s1, Figure S1: Body Composition Measurement using Fresenius Bioelectrical Impedance Spectroscopy. Table S1: Criteria Proposed by ISRNM to Diagnose PEW, Figure S2: Flowchart of Patient Recruitment, Figure S3: Other Major Comorbidities, Table S2: Comparison of PhA across Patients’ Characteristics, Table S3: Correlations between PhA with PEW Criteria and Body Composition in HD Patients, Figure S4: Overall Model Quality, Table S4: Comparisons of Previous Studies using PhA for PEW Diagnosis, Table S5: Sub-group Analysis for Diagnostic Accuracy of PhA according to Ethnicity to detect PEW in HD patients.
