# Supplementary material for: Bioelectrical Impedance Analysis Derived-Phase Angle as a Pragmatic Tool to Detect Protein Energy Wasting among Multi-Ethnic Hemodialysis Patients
Source: Diagnostics (Basel). 2021 Sep 23;11(10):1745. doi: 10.3390/diagnostics11101745 (PMC8534349; doi:10.3390/diagnostics11101745)

Supplementary Material Figure S1. Body Composition Measurement using Fresenius Bioelectrical Impedance Spectroscopy.

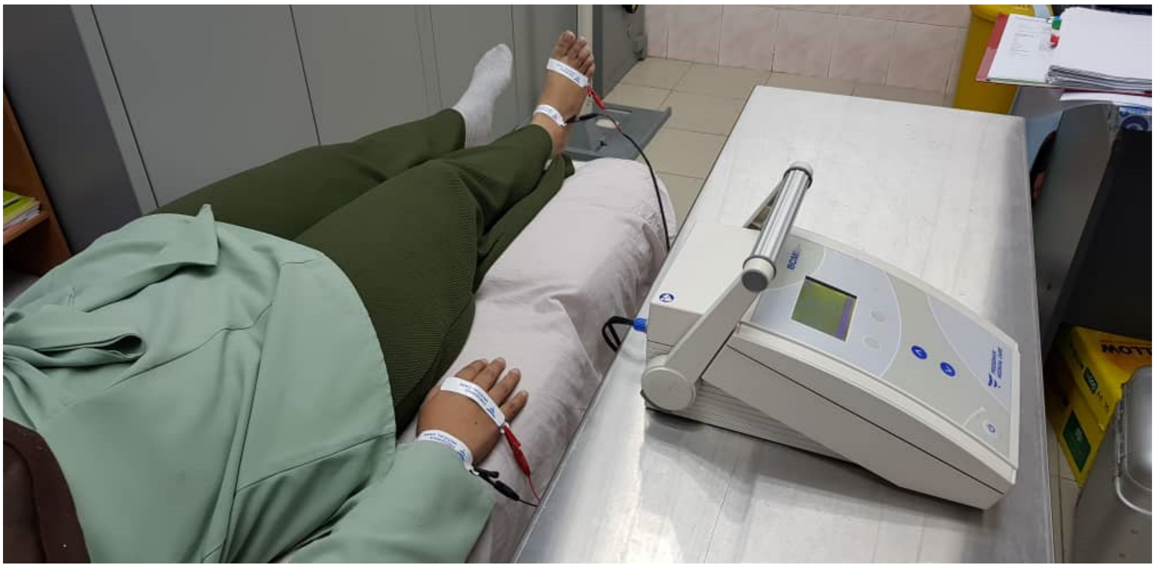

Supplement: Supplementary file 1 [file diagnostics-11-01745-s001.zip › Supplementary Material Figure S1.pdf]
