# Supplementary material for: Bioelectrical Impedance Analysis Derived-Phase Angle as a Pragmatic Tool to Detect Protein Energy Wasting among Multi-Ethnic Hemodialysis Patients
Source: Diagnostics (Basel). 2021 Sep 23;11(10):1745. doi: 10.3390/diagnostics11101745 (PMC8534349; doi:10.3390/diagnostics11101745)
Supplement: Supplementary file 1 [file diagnostics-11-01745-s001.zip › Supplementary Material Figure S3.pdf]

Supplementary Material Figure S3. Other Major Comorbidities (n = 152).

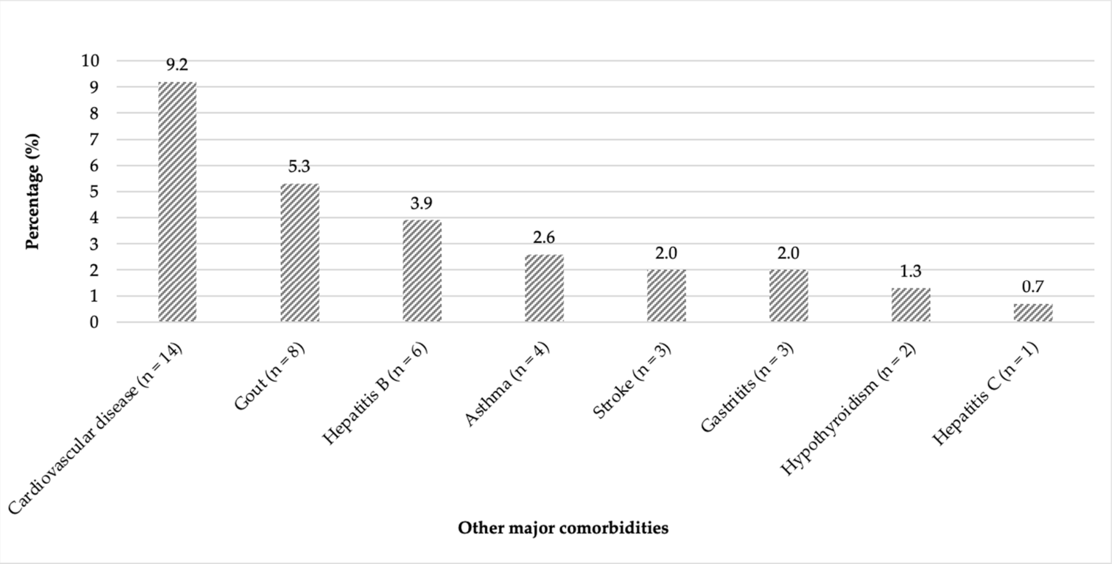

Figure S3. The frequency for other major comorbidities does not equal to the total number of patients because some patients have multiple comorbidities.
