# Supplementary material for: Bioelectrical Impedance Analysis Derived-Phase Angle as a Pragmatic Tool to Detect Protein Energy Wasting among Multi-Ethnic Hemodialysis Patients
Source: Diagnostics (Basel). 2021 Sep 23;11(10):1745. doi: 10.3390/diagnostics11101745 (PMC8534349; doi:10.3390/diagnostics11101745)
Supplement: Supplementary file 1 [file diagnostics-11-01745-s001.zip › Supplementary Material Table S1.pdf]

Supplementary Material Table S1. Criteria Proposed by ISRNM to Diagnose PEW.

Criteria
**1. Body weight and fat (body mass)**
Body mass index < 23kg/m^2;
Body fat percentage < 10%;
Unintentional weight loss over time: 5% over 3 months or 10% over 6 months.
**2. Muscle mass**
Mid-arm muscle circumference: reduction > 10% in relation to 50th percentile of reference population;
Reduced muscle mass: 5% over 3 months or 10% over 6 months.
**3. Serum chemistry**
Serum albumin < 38 g/L;
Serum cholesterol < 2.59 mmol/L.
**4. Dietary intake**
Unintentional low dietary protein intake < 0.8 g/kg BW/day for 2 months;
Unintentional low dietary energy intake < 25 kcal/kg BW/day for 2 months.

Source: [2] Fouque et al.
