# Supplementary material for: Bioelectrical Impedance Analysis Derived-Phase Angle as a Pragmatic Tool to Detect Protein Energy Wasting among Multi-Ethnic Hemodialysis Patients
Source: Diagnostics (Basel). 2021 Sep 23;11(10):1745. doi: 10.3390/diagnostics11101745 (PMC8534349; doi:10.3390/diagnostics11101745)
Supplement: Supplementary file 1 [file diagnostics-11-01745-s001.zip › Supplementary Material Table S2.pdf]

Supplementary Material Table S2. Comparison of PhA across Patients' Characteristics (n = 152).

Variable	PhA (°)	*p*-Value
Age (years)		<0.001
18-30	5.17 ± 0.71 [a]	
31-40	4.91 ± 0.82 [b]	
41-50	4.75 ± 0.83 [c]	
51-60	4.31 ± 0.92	
> 60	3.93 ± 0.81 [abc]	
Gender		<0.001
Male	4.62 ± 0.82 [a]	
Female	3.92 ± 0.88 [a]	
Ethnicity		
Malay	4.49 ± 0.96 [a]	0.012
Chinese	4.04 ± 0.85 [a]	
Indian	4.07 ± 0.68	
Education level		0.024
Primary	3.98 ± 0.77 [a]	
Secondary	4.35 ± 0.96	
Tertiary	4.52 ± 0.92 [a]	
Marital status		0.094
Single	4.65 ± 0.99	
Married	4.25 ± 0.90	
Employment		<0.001
Employed	4.75 ± 0.93 [a]	
Unemployed	4.13 ± 0.86 [a]	
Monthly income		0.051
≤ RM 1000	4.14 ± 0.85	
> RM 1000	4.43 ± 0.96	
No of comorbidities		0.020
None	4.34 ± 1.03	
One	4.59 ± 0.96 [a]	
Two	4.17 ± 0.81	
≥ Three	4.03 ± 0.86 [a]	
Dialysis vintage (months)		0.356
< 12	4.38 ± 1.37	
12-48	4.41 ± 0.87	
> 48	4.19 ± 0.91	
Dialysis adequacy (Kt/V)		0.897
< 1.2	4.27 ± 0.77	
≥ 1.2	4.30 ± 0.95	

Data are expressed as mean ± SD; Data were analysed using independent t-test and one-way ANOVA;
[a,b,c] Data shared the same superscript indicates that they are significant from each other.
