# Supplementary material for: Bioelectrical Impedance Analysis Derived-Phase Angle as a Pragmatic Tool to Detect Protein Energy Wasting among Multi-Ethnic Hemodialysis Patients
Source: Diagnostics (Basel). 2021 Sep 23;11(10):1745. doi: 10.3390/diagnostics11101745 (PMC8534349; doi:10.3390/diagnostics11101745)
Supplement: Supplementary file 1 [file diagnostics-11-01745-s001.zip › Supplementary Material Table S3.pdf]

Supplementary Material Table S3. Correlations between PhA with PEW Criteria and Body Composition in HD Patients (n = 152).

**Categories**	***r***	***p*-Value**
Body weight and fat (body mass)		
BMI (kg/m^2)	0.175	0.031
BF (%)	-0.382	<0.001
Unintentional weight loss (%)	0.093	0.255
Muscle mass		
MAMC (cm)	0.444	<0.001
Serum creatinine (umol/L)	0.542	<0.001
Serum chemistry		
Albumin (g/L)	0.283	<0.001
Cholesterol (mmol/L)	0.209	0.010
Dietary data		
DEI (kcal/kg BW/day)	0.074	0.362
DPI (g/kg BW/day)	0.057	0.484
Body Composition		
OH (L)	-0.420	<0.001
LTI (kg/m^2)	0.718	<0.001
FTI (kg/m^2)	-0.160	0.048
ICW (L)	0.658	<0.001
ECW (L)	0.251	0.002
BCM (kg)	0.690	<0.001

Data was analysed using Pearson Product-Moment Correlation; Abbreviations: BMI, body mass index; BF, body fat; MAMC, mid-arm muscle circumference; DEI, dietary energy intake; DPI, dietary protein intake; BW, body weight; OH, overhydration; LTI, lean tissue index; FTI, fat tissue index; ICW, intracellular water; ECW, extracellular water; BCM, body cell mass.
