# Supplementary material for: Bioelectrical Impedance Analysis Derived-Phase Angle as a Pragmatic Tool to Detect Protein Energy Wasting among Multi-Ethnic Hemodialysis Patients
Source: Diagnostics (Basel). 2021 Sep 23;11(10):1745. doi: 10.3390/diagnostics11101745 (PMC8534349; doi:10.3390/diagnostics11101745)
Supplement: Supplementary file 1 [file diagnostics-11-01745-s001.zip › Supplementary Material Table S4 .pdf]

**Supplementary Material Table S4.** Comparisons of Previous Studies using PhA for PEW Diagnosis.

| Author | Country | Study Design | Sample Size | Age (years) | Female, n (%) | BIA Model | BIA Type | PhA Cut-Off (°) | AUC | Sensitivity (%) | Specificity (%) |
|---|---|---|---|---|---|---|---|---|---|---|---|
| Ruperto et al., 2014 | Spain | Cross-sectional | 80 | 68.0 ± 14.4 | 28 (35.0) | BIVA-101 | Single frequency | 4.00 | NA | NA | NA |
| Tan et al., 2019 | China | Cross-sectional | 173 | 58.0 ± 11.8 | 78 (45.1) | InBody S10 | Multi-frequency | 4.60 | 0.870 | 86.4 | 76.3 |
| Leal-Escobar et al., 2019 | Mexico | Cross-sectional | 69 | 36.5 ± 13.4 | 36 (52.0) | InBody S10 | Multi-frequency | 4.64 | 0.776 | 77.7 | 76.1 |
| Saitoh et al., 2020 | Japan | Retrospective cohort | 116 | 64.0 ± 12.0 | 41 (35.0) | Seca mBCA515 | Multi-frequency | 3.70 | 0.718 | NA | NA |
| Zhou et al., 2021 | China | Cross-sectional | 64 | 59.6 ± 12.6 | 31 (48.4) | InBody 770 | Multi-frequency | 4.45 | 0.749 | 83.3 | 37.5 |

Abbreviations: BIA, bioelectrical impedance analysis; AUC, area under the curve; NA, not available
