# Supplementary material for: Bioelectrical Impedance Analysis Derived-Phase Angle as a Pragmatic Tool to Detect Protein Energy Wasting among Multi-Ethnic Hemodialysis Patients
Source: Diagnostics (Basel). 2021 Sep 23;11(10):1745. doi: 10.3390/diagnostics11101745 (PMC8534349; doi:10.3390/diagnostics11101745)
Supplement: Supplementary file 1 [file diagnostics-11-01745-s001.zip › Supplementary Material Table S5.pdf]

Supplementary Material Table S5. Sub-group Analysis for Diagnostic Accuracy of PhA according to Ethnicity to detect PEW in HD Patients (n = 152).

Ethnicity	PhA Cut-Off (°)	adjAUC	Sensitivity (%)	Specificity (%)	*p*-Value
Malay (n = 84)	4.26	0.744	64.3	60.0	<0.001
Chinese (n = 50)	3.85	0.692	61.5	67.6	0.028
Indian (n = 18)	4.14	0.785	80.0	53.8	0.009

Abbreviations: PhA, phase angle; adjAUC, area under the curve adjusted for age, dialysis vintage, comorbidities, measurement quality, and overhydration. Data was analysed using Receiver Operating Characteristics curve analysis.
