# Supplementary figures and images for: Bioelectrical Impedance Analysis Derived-Phase Angle as a Pragmatic Tool to Detect Protein Energy Wasting among Multi-Ethnic Hemodialysis Patients
Source: Diagnostics (Basel). 2021 Sep 23;11(10):1745. doi: 10.3390/diagnostics11101745 (PMC8534349; doi:10.3390/diagnostics11101745)

**Supplementary Material Figure S2. Flowchart of Patient Recruitment.**

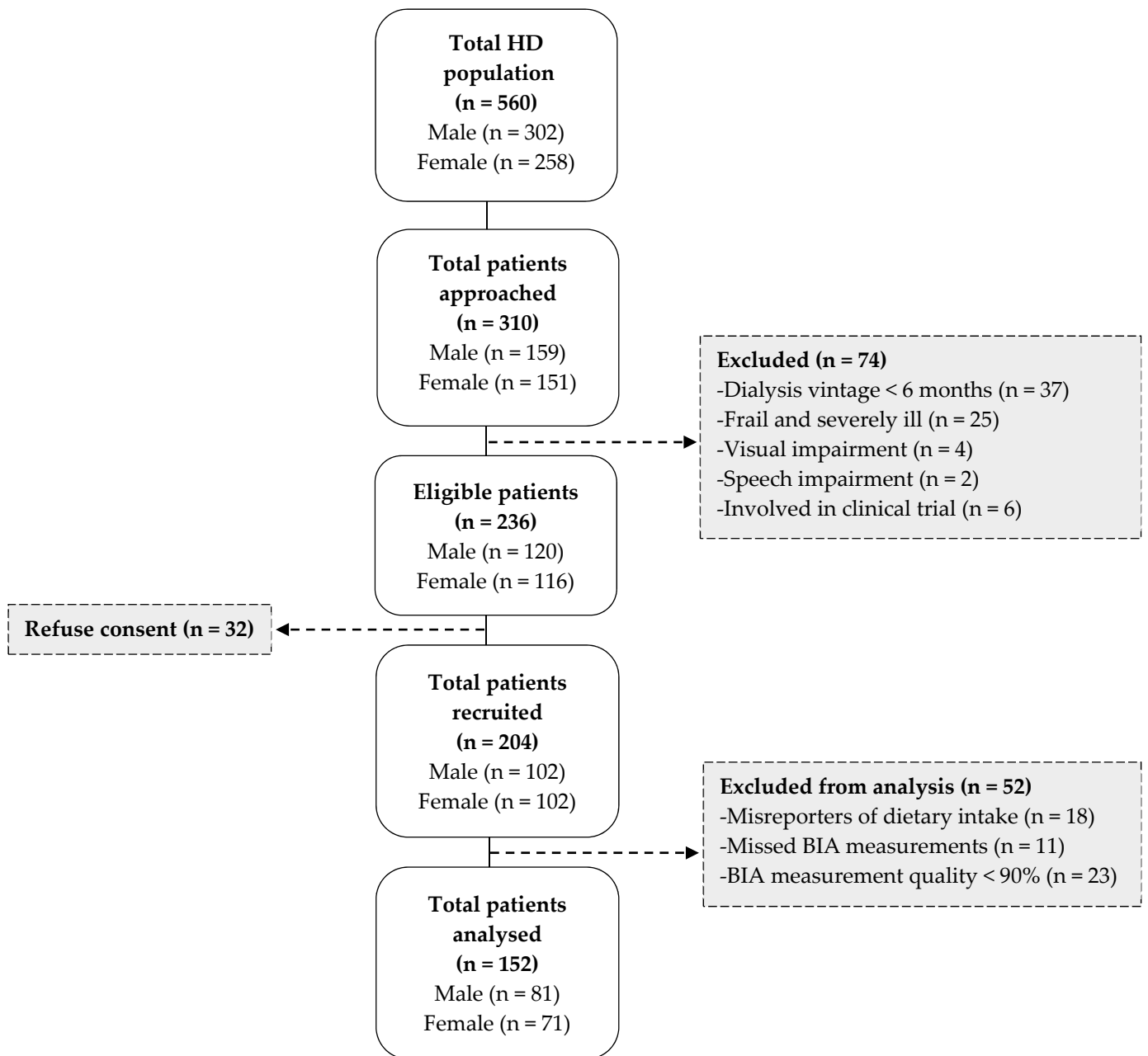

Supplement: Supplementary file 1 [file diagnostics-11-01745-s001.zip › Supplementary Material Figure S2.pdf]

Supplementary Material Figure S3. Overall Model Quality.

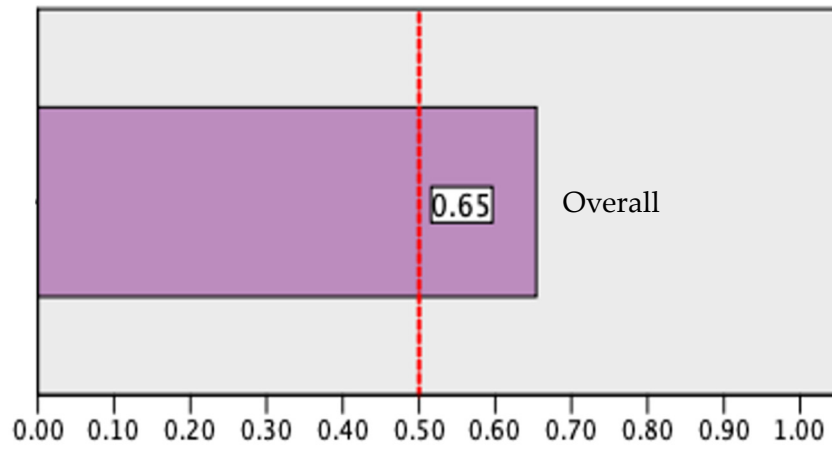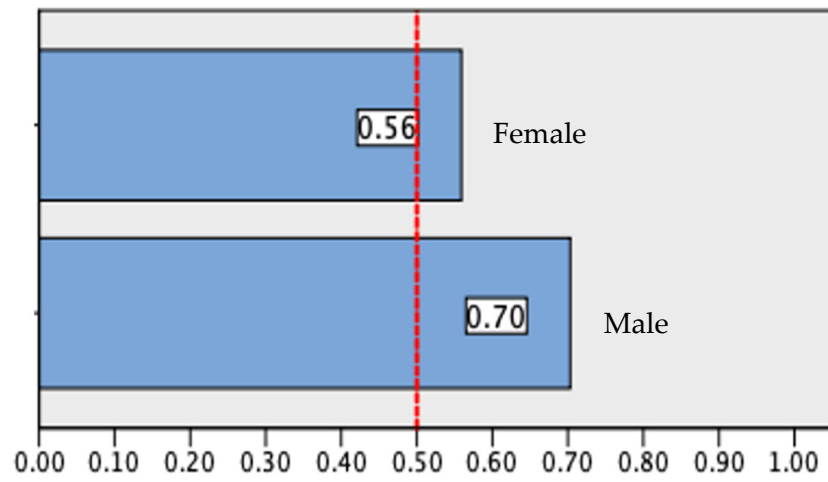

Supplement: Supplementary file 1 [file diagnostics-11-01745-s001.zip › Supplementary Material Figure S4.pdf]
